# Hygrothermal Ageing Influence on BVI-Damaged Carbon/Epoxy Coupons under Compression Load

**DOI:** 10.3390/polym13132038

**Published:** 2021-06-22

**Authors:** Maria Pia Falaschetti, Matteo Scafé, Nicola Zavatta, Enrico Troiani

**Affiliations:** 1 Department of Industrial Engineering DIN, University of Bologna, Via Fontanelle 40, 47121 Forlì, Italy; nicola.zavatta2@unibo.it (N.Z.); enrico.troiani@unibo.it (E.T.); 2 Laboratorio Tecnologie dei Materiali Faenza, ENEA-SSPT-PROMAS-TEMAF, Via Ravegnana 186, 48018 Faenza, Italy; matteo.scafe@enea.it

**Keywords:** composite materials, compressive strength, impacts, BVID, hygrothermal ageing

## Abstract

Composite materials usage in several industrial fields is now widespread, and this leads to the necessity of overcoming issues that are still currently open. In the aeronautic industry, this is especially true for Barely Visible Impact Damage (BVID) and humidity uptake issues. BVID is the most insidious kind of impact damage, being rather common and not easily detectable. These, along with the ageing that a composite structure could face during its operative life, could be a cause of fatal failures. In this paper, the influence of water absorption on impacted specimens compressive residual strength was studied. Specimens were impacted using a modified Charpy pendulum. Two different locations were chosen for comparison: Near-Edge (NE) and Central (CI). Accelerated hygrothermal ageing was conducted on impacted and reference nonimpacted coupons, placing them in a water-filled jar at 70 °C. Compressive tests were performed in accordance with the Combined Loading Compression (CLC) test method. A Dynamic Mechanical Analysis (DMA) was performed as well. The results showed the influence of hygrothermal ageing, as expected. Nevertheless, the influence of impact location on compressive residual strength is not clearly noticeable in aged specimens, leading to the conclusion that hygrothermal ageing may have a greater effect on composite compressive strength than the analysed BVI damage.

## 1. Introduction

Water absorption is a significant issue for advanced composite materials due to its significant effect on physical, mechanical, and chemical properties. Matrix-conditioned properties, such as compressive and interlaminar shear strength, are affected by hygrothermal ageing [1]. This is due to the plasticisation of the polymer chains [2,3,4,5,6,7], easily detected by measuring the material glass transition temperature (T_g_) by means, for example, of the Dynamic Mechanical Analysis (DMA). Water absorption, moreover, could degrade the fibre/matrix interface, resulting in a decrease of stress transfer and in a microcracking phenomenon that happens when an uneven swelling of the matrix occurs [2,5,8,9,10]. Many parameters affect water uptake: the environmental temperature and percentage of humidity, matrix system, matrix/fibre interface, fibre material, fibre surface roughness, defects, etc. Due to these, water absorption could hence be caused by means of diffusion and capillary flow mechanisms [11,12,13]. Capillary flow takes advantage of voids, defects, microcracks, interface flaws. Moreover, high fibre volume fractions could facilitate this process. Diffusion, on the other hand, depends on chemical bonds and molecular disposition. Thermoset resins are hydrophilic materials due to their chemical structure. In particular, epoxy resins, which have a high concentration of hydrogen bonds, are subjected to a higher moisture uptake compared to other thermoset resins, like vinyl ester and urethane acrylate [14].

The fibre/matrix interfacial integrity and properties are essential for composite performances. Different fibre/matrix combinations, fibre surface roughness, and matrix fillers could condition the interface resistance to moisture sensitively. Due to the great importance of environmental issues, many studies have been carried out on vegetable composite reinforcements. Due to their hydrophilic nature, these materials are quite affected by hygrothermal ageing [15], even in a rather complex way. For instance, wood flour filler, despite being a cause of weakening in the early stages of moisture absorption, could improve the resin/fibre interface behaviour near the saturation condition [16]. Moreover, matrix swelling could reduce the transverse residual strains caused by curing [17].

Water uptake is primarily dependent on the moisture percentage that the composite material is exposed to. Diffusion rates are higher as the environmental moisture content increases. Consequently, the mechanical characteristics vary considerably depending on the operating conditions [7]. Immersion in water has been widely used as a standard procedure for the purpose of studying moisture absorption in different humid environments in an accelerated way [18].

The environmental temperature is also an important parameter. The mechanical performances of composite materials are affected by high temperatures. The glass transition temperature of the matrix (T_g_) is a clear dividing line: in operating conditions below T_g_, the strength decreases steadily as the temperature increases; beyond it, the matrix shows a rubbery behaviour; at higher temperatures (>500 °C), the fibres could incur oxidation or decomposing processes, resulting in an even greater drop in performances [19,20,21]. For moisture absorption tests, temperatures below T_g_ can be safely applied to boost moisture absorption [22] while avoiding any possible influence on the chemical characteristics of the composite.

The main failure mechanisms of composite materials are delamination, matrix cracking, and fibre breakage [23,24,25]. When used advantageously, (e.g., in crash absorbers [26,27,28,29,30]) these components can offer a significant energy absorption capacity. The brittle nature of the material, however, can be troublesome when a component is subjected to impact by foreign objects, like running debris or tools [23,31]. Impact damage is, in fact, detrimental to the mechanical performances of structures. This issue regards not only thermoset resins but also thermoplastic and geopolymer matrix systems [32,33,34].

In the aeronautical industry, hygrothermal ageing has gained interest due to the widespread use of composite materials, especially Carbon Fibre Reinforced Polymers (CFRP), in secondary and primary structures. Moreover, aeronautical structures are quite exposed to foreign objects impacts. This results in defects on the surfaces, like micro-cracks, which could facilitate water absorption and consequent material degradation [35,36].

In an airplane lifetime, Barely Visible Impact Damage (BVID) is considered the most deceitful kind of impact damage. BVID is rather common and not detectable with usual visual inspections [37,38]. In fact, it could result in wide damage beyond the surface without any clear evidence on the outside. Nowadays, Non-Destructive Inspection (NDI) techniques are extensively used thanks to the possibility to locate the damage and take proper maintenance decisions [39,40,41]. However, full-scale structure NDI is still challenging. Understanding how BVID leads to the decrease of the mechanical characteristics of structures [25,38,42] is hence beneficial.

In [43], the authors demonstrated that material compressive residual strength was influenced by the impact location. 3 and 5 J impacts were performed on a cross-ply carbon/epoxy laminate, and two different locations were chosen: central and near-edge. Specimens were then tested by means of the Combined Loading Compression (CLC) test method. All impacted specimens showed a drop in compressive residual strength, but, while the central impact had a similar decrease for both energy values (9.9% for 3 J and 14.1% for 5 J), near-edge impacted coupons showed more noticeable reductions: compared with the reference material, a 31.2% decrement was measured for the 5 J near-edge impact. Moreover, it was noticed that while the central impact could result in cracks on the structure surface around the impact indentation only, a near-edge impact could lead to through-the-thickness cracks. In the presence of humidity, this phenomenon could worsen the moisture absorption [35,36] and therefore lead to a greater decrease of mechanical strength.

The aim of this paper is to investigate how BVI damage on a carbon/epoxy laminate could be influenced by hygrothermal ageing. Different impact locations (central and near-edge) and hygroscopic ageing times (up to 45 days) have been studied. The mechanical, physical, and chemical effects of accelerated hygrothermal ageing on BVI-damaged carbon/epoxy specimens have been investigated.

## 2. Materials and Methods

### 2.1. Tested Materials

A carbon/epoxy unidirectional prepreg, Deltapreg UTS-300-DT120-37EF [44], was tested. This is a unidirectional 300 gsm High Strength (HS) carbon fibres tape impregnated with a high toughness resin. This resin system has good impact resistance and is therefore particularly used for structural applications. The laminate consisted of 13 plies with a cross-ply, balanced, and symmetric stacking sequence ${\left[90/0_{2}/90/0/90/\bar{90}\right]}_{s}$ (the overline denotes the central ply). It was assembled by means of hand lay-up. The parameters of the autoclave cure cycle were chosen in agreement with the manufacturer’s instructions (Delta Preg, Sant’Egidio alla Vibrata, Italy): the laminate was cured at 120 °C and 5 bar for 90 min (heating and cooling rates equal to 2 °C/min).

The specimens were cut from the cured laminate by means of a Cortini CNC milling machine (Fidia Group, Forlì, Italy), with the following dimensions:

- compression specimens: 140 mm × 30 mm × 4.2 mm,
- DMA coupons: 50 mm × 5.6 mm × 4.2 mm.

### 2.2. Impact Tests

Impact tests were performed by means of a modified Charpy pendulum assembled at the Hangar Laboratories of University of Bologna.

The specimens length was chosen in accordance with ASTM D6641/D6641M-16 [45], while the width was chosen as the maximum allowable, compatible with the CLC test fixture. This choice also allowed one to perform both kinds of impacts on the same geometry, avoiding the influence of free edges on central impacts. ASTM D6641 does not specify a limit for specimen thickness, except the implicit requirement to avoid buckling during tests.

**Figure 1:**
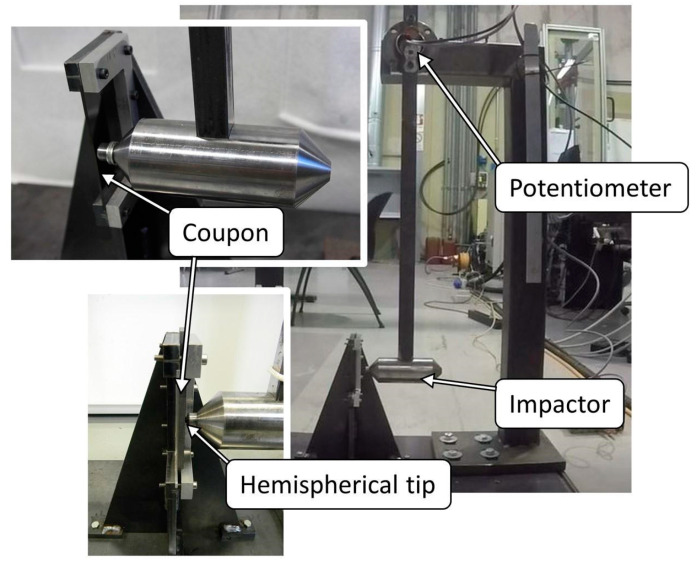
Equipment used for the impact tests (Modified Charpy pendulum).

**Figure 2:**
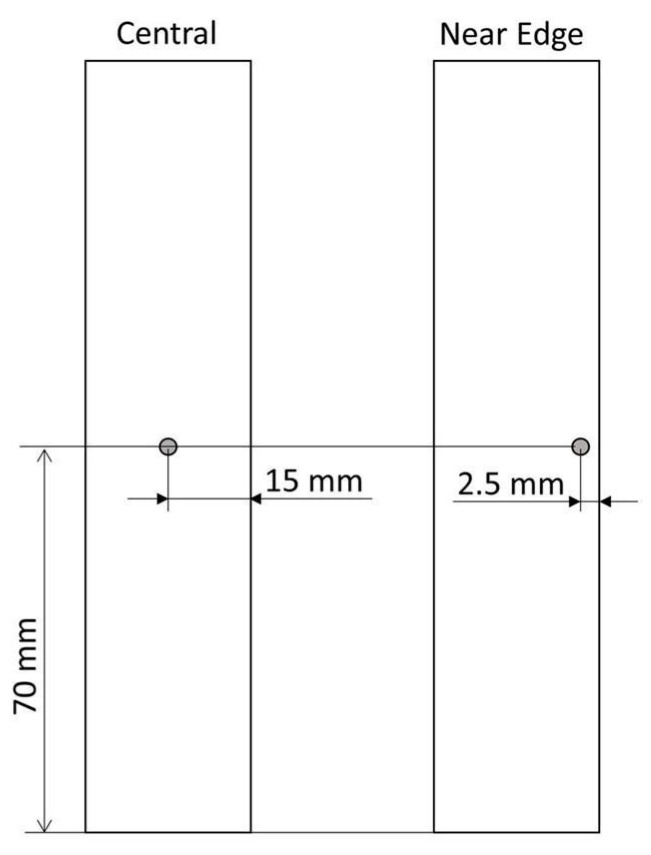
Impact locations.

The swinging mass is a steel cylinder with a 7 mm hemispherical impactor (Figure 1). During the impact test, the specimen is held in position to obtain an impact orthogonal to the coupon surface. Two conditions are tested: central impacts (CI) and near-edge impacts (NE). In central impacts, the specimen is impacted at half of its width, while in near-edge impacts it is impacted at a distance of 2.5 mm from the edge. As shown in Figure 2, in both cases impacts take place in the middle of the specimen length, which corresponds to the centre of the CLC gauge section.

The pendulum swinging mass support is connected to a potentiometer that allows the measurement of the release angle of the mass and the rebound angle. Therefore, the impact energy and rebound energy were calculated by means of (1):

(1) E=mgl (1−cos α)

where E is the energy, m the impactor mass, g the gravitational acceleration, l the pendulum length, and α the angle (initial or rebound). The absorbed energy was calculated through (2):

(2) $$E_{ab}=E_{in}-E_{res}$$

where $E_{ab}$ is the absorbed energy, $E_{in}$ is the initial energy (3 J in all tests), and $E_{res}$ is the residual energy in the impactor.

### 2.3. Accelerated Ageing and Mass Variation

After the impact tests, compression and DMA coupons were placed inside closed jars, filled with water, and put in an oven at 70 °C to perform accelerated hygrothermal ageing, in accordance with [18].

Five different ageing periods were considered: 0 (unaged material as reference), 1 day, 3 days, 7 days, and 45 days. The mass variation (M [%]) was calculated by means of Equation (3) [18]:

(3) $$M=\left|\frac{W_{end}-W_{in}}{W_{in}}\right|*100$$

where $W_{end}$ is the final mass in [g], and $W_{in}\;$is the initial mass in [g].

$W_{end}$ and the specimens post-ageing dimensions were measured 30 min after they had been removed from the oven, avoiding the influence of extra water and coupons swelling due to temperature. Moreover, the specimens were weighed and measured again the day after, in order to detect any changes: the coupons were left to dry naturally on a clean surface, far from UV rays or forced air currents.

### 2.4. DMA Tests

Fifteen coupons for DMA analysis were cut from the same laminate used to obtain the compression test specimens and were processed with the accelerated ageing described previously. Three coupons per ageing time were tested by means of a NETZSCH DMA 242 E Artemis (NETZSCH Gruppe, Selb, Deutschland) at University of Bologna laboratories under single-cantilever bending [46].

The applied sinusoidal load had a 6 N amplitude at 1 Hz frequency and a maximum displacement of 10 μm. The heating rate was 3 °C/min. The test started at room temperature, and each specimen was tested up to 150 °C, the expected tanδ being below this value.

### 2.5. CLC Tests

Compression tests were performed by means of a Combined Loading Compression (CLC) (Wyoming Test Fixtures Inc., Salt Lake City, UT, USA) test fixture [45,47]. The experimental tests were conducted at ENEA TEMAF Laboratories using an MTS servo-hydraulic universal testing machine equipped with an MTS 100 kN load cell (MTS Systems Corporation, Eden Prairie, MN, USA). All tests were performed at constant displacement rate (1.3 mm/min), while data were acquired at 10 Hz and processed in accordance with the ASTM standard.

The compressive residual strength was calculated by means of Equation (4):

(4) $$\sigma =\frac{F}{A}$$

where F is the load, A is the nominal cross-section of the specimen, and σ is the residual strength.

### 2.6. Micrographs

Microscopy specimens (aged and reference) were embedded in epoxy resin so as to hold them during polishing. The lapping steps started with 600 grit grinding paper, up to 1 μm diamond particles. Micrographic pictures were taken by means of a Nikon OPTIPHOT-100 microscope (Nikon Instruments Spa, Campi Bisenzio, Italy) up to a magnification of 50×. The scope of this analysis was to detect any macroscale alteration in the resin and fibre-resin interface appearances.

## 3. Results and Discussion

### 3.1. Impact Tests Results

In Table 1, the mean values of the impact tests are listed.

**Figure 3:**
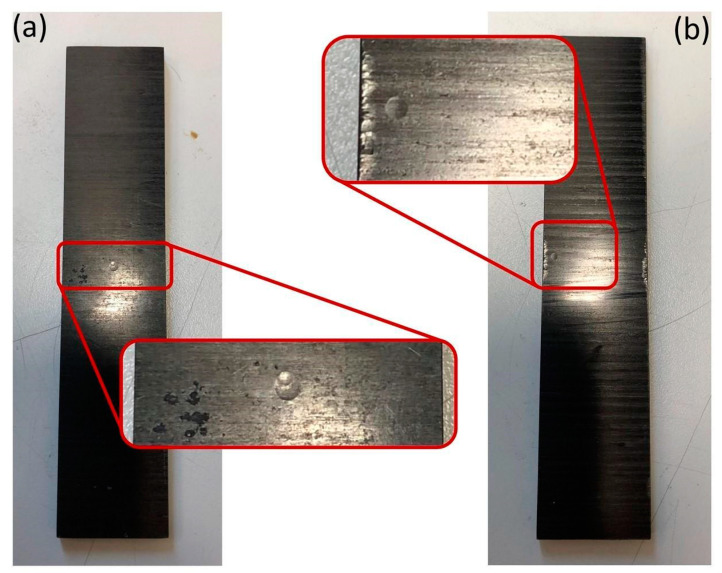
Examples of different impact locations: (**a**) central impact and (**b**) near-edge impact.

In Figure 3, two examples of central and near-edge impact indentations are shown. The indentations are barely visible, but they can be located by means of a proper light.

### 3.2. Accelerated Ageing and Absorption Data

**Figure 4:**
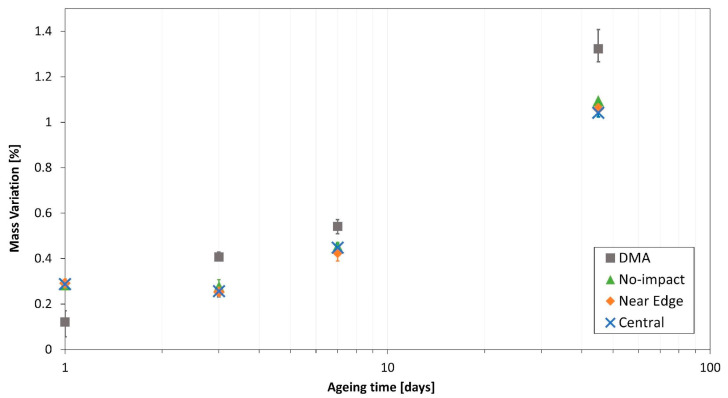
Mass variations mean values comparison—all coupons. A logarithmic scale was used for the horizontal axis.

The accelerated ageing procedure led to an increase of the coupons mass due to water absorption, which was higher as the ageing time increased. In Figure 4, the mass variation of all compression and DMA specimens is shown.

Comparing CLC and DMA specimens mass variations, it is noticeable that CLC specimens tend to have a lower increase than DMA specimens. Furthermore, CLC specimens showed the same behaviour, independently of the impact location. For further comprehension, the mean mass variation values are listed in Table 2.

When analysing the weight values, it was noticed that 24 h after removal from the oven no further mass changes had taken place. Therefore, all mass variations related to these measurements are not listed.

### 3.3. Effect of Ageing on T_g_

**Figure 5:**
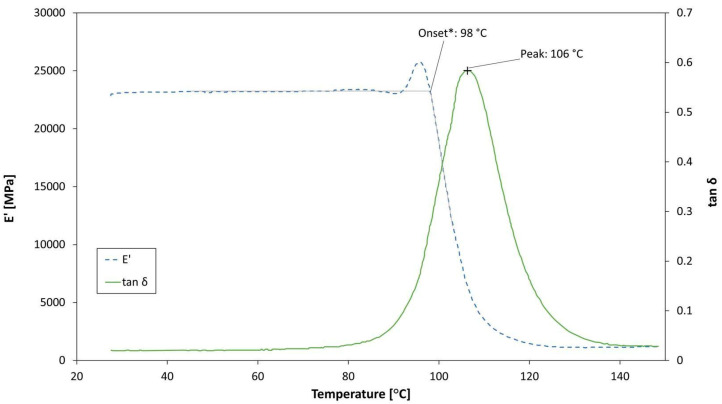
DMA results example—7 days aged coupons.

In Figure 5, an example of the DMA test curve is plotted. Table 3 lists the measured glass transition temperatures (T_g_ measurements are determined considering the tanδ peak). The unaged material showed a T_g_ consistent with the data provided by the manufacturer (115–120 °C for 90′ @120 °C cure cycle [44]), while aged coupons resulted in a lower glass transition temperature. Hygroscopic ageing, therefore, resulted in a plasticisation of the resin, due to polymer chains scission that increased the chains mobility. This behaviour is consistent with previous studies in the literature [2,3,4,5,6,48,49].

### 3.4. Effect of Ageing on Compressive Behaviour

Three no-impact, five near-edge impact, and five central impact specimens were tested under compression for each ageing time.

**Figure 6:**
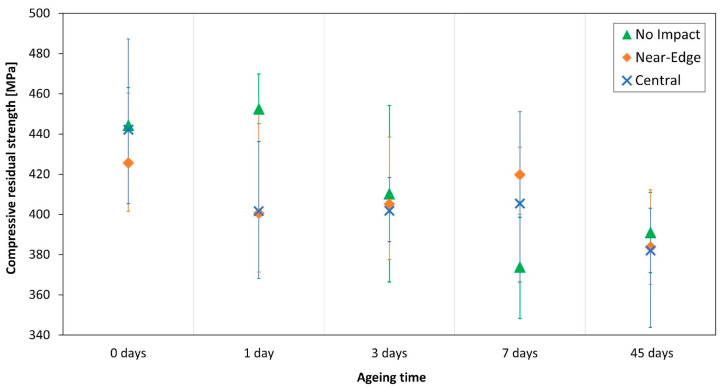
Compressive residual strength.

As shown in Figure 6, a slight decrease in compressive residual strength, for the same impact location, takes place as the ageing time increases. Although, as usual for composite materials, data scatter is not negligible (Table 4), a decreasing trend is nevertheless identifiable.

In the early stages of ageing, i.e., 1 day, the impacted specimens show a lower compressive residual strength than the no-impact specimens. This effect is more relevant for near-edge impacted coupons. Moreover, impacted specimens exhibited a drop in strength with increased ageing. However, as the ageing time rises, this effect decreases. Similarly, when comparing the compressive residual strength at an ageing time beyond 1 day, a clear effect from different impact locations is not observed.

In Table 5, the compressive residual strength percentage variations are listed: these values are computed to analyse the impact location effect within the same ageing time (Impact Δσ) and the effect of the ageing time within the same impact location (Ageing Δσ).

Analysing these percentage variations, the previous inferences are confirmed: the influence of the ageing time on the compressive residual strength is evident for all the impact locations, but, concurrently, the impact location does not influence the material compressive residual strength for the same ageing time (from 1 day on). This outcome is apparently in contrast with previous experiences [25,43], but an explanation could be found in the data scatter due to the very low energy of the impacts.

**Figure 7:**
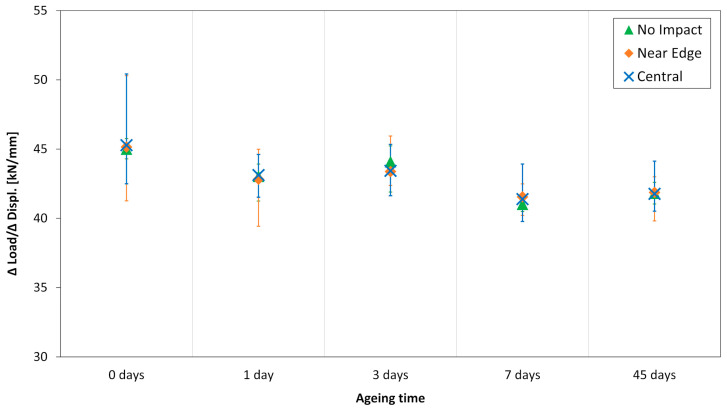
Specimen stiffness at different ageing times.

Due to the presence of impact indentations in the middle of the gauge section, it was preferred not to install strain gauges on the specimen surface. Consequently, the strain was not measured. However, the effect of ageing on specimen stiffness was assessed as follows. For each specimen, the slope of the linear part of the load-displacement curve was calculated and used as a basis for evaluating the stiffness. The computed values are shown in Figure 7.

**Figure 8:**
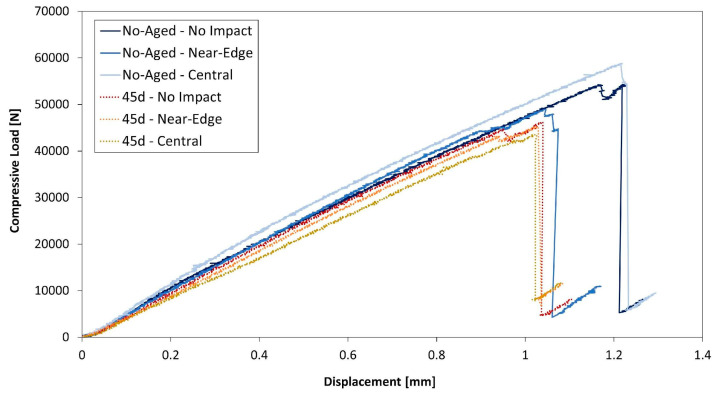
Load-displacement curves. Unaged (solid line) and 45 day-aged (dotted lines) specimens, for all impact locations.

As for the compressive strength, the specimen stiffness exhibited an evident data scatter. However, a decreasing trend could be identified. In the case of the 45 days aged specimens, the stiffness is about 7.5% lower than the unaged specimens, confirming the effect of the hygrothermal ageing. On the other hand, there is no clear influence of the impact location. Additionally, the slight decrease in the 45 days specimens stiffness is visible looking at the load-displacement curves in Figure 8.

### 3.5. Micrographs

**Figure 9:**
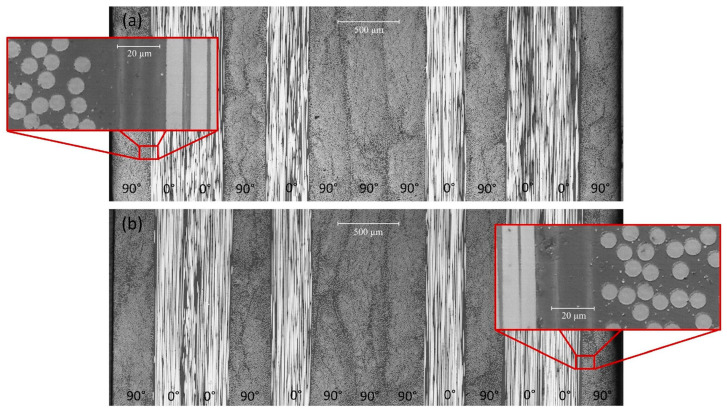
Micrograph picture (5×): (**a**) unaged material, (**b**) 45 days aged material. The whole stacking sequence is shown. 50× details are shown for both ageing times.

In Figure 9, 5× micrographs of unaged and 45 days aged material are shown: a small swelling can be observed. Moreover, 50× details are presented. Comparing these higher magnification pictures, in the aged material a small shadow can be pinpointed around the fibres border, while the matrix appears damaged close to the [0°] ply. These effects could be attributed to the softening effect of hygroscopic ageing on the resin [50]. A higher magnification is needed for a deeper investigation of this outcome.

## 4. Conclusions

In this paper, the influence of hygrothermal ageing on impacted composite material was investigated. CFRP coupons (cut in accordance with the requirements of the Combined Loading Compression test method) were impacted with an energy of 3 J by means of a modified Charpy pendulum. Accelerated ageing was performed by immersing the coupons in water at 70 °C. Different ageing times were considered. As a reference, some specimens were tested as built.

DMA tests were conducted to measure the Glass Transition temperature of reference and aged materials. These tests confirmed the expected ageing influence on material characteristics and, therefore, the plasticisation of the epoxy resin due to water absorption. Moisture absorption data showed a weight gain for all the specimens. A slightly higher uptake could be pointed out for the DMA coupons, while all impacted specimens showed almost the same behaviour. The impact location, hence, seems to have no influence.

Micrographic analysis revealed a slight swelling of the coupons. Comparing magnification pictures up to 50×, the aged material presents small shadows around the fibres border, while the matrix appears damaged close to the [0°] ply. These effects could be put down to the resin softening due to hygroscopic ageing.

CLC tests were conducted to measure the residual compressive strength of the material. The results showed the influence of the ageing time on all the coupons. Additionally, the ageing overcame the effect of the impact location on the residual strength (for the same ageing time). Notwithstanding this, the impact location effect is noticeable in the unaged group, revealing a higher decrease of the compressive residual strength for the near-edge- rather than for the central-impacted coupons. Moreover, comparing the unaged and 1 day groups, a noticeable decrease in the residual strength of the impacted specimens was found. Furthermore, when analysing the slope of the load-displacement curves, the stiffness of the 45 days aged specimens exhibited a slight decrease compared to the unaged specimens, confirming the effect of ageing on the material. As usual for composite materials, data scatter is not negligible: a greater statistical sample may be beneficial in obtaining more defined results.

In conclusion, Barely Visible Impact Damage is sensitive to impact location. A low-energy impact near the edge could result in the impairment of component performances, even more so when a humid environment is involved. Nevertheless, when hygrothermal ageing is severe, its consequences on composite strength may be more serious than those of a low-energy (3 J) impact.

## Figures and Tables

**Table 1:** Impact energies.

Impact Area		Impact Energy [J]	Residual Energy [J]	Absorbed Energy [J]
Central	Mean Value	3.03	0.21	2.82
	Std Dev.	0.02	0.05	0.05
Near-Edge	Mean Value	3.03	0.33	2.70
	Std Dev.	0.02	0.03	0.03

**Table 2:** Mass variation mean values comparison.

Ageing Time [Days]	Mass Variation [%]—Mean Values			
	No-Impact	NE	CI	DMA
0	0	0	0	0
1	0.29	0.29	0.29	0.12
3	0.28	0.25	0.26	0.41
7	0.45	0.43	0.45	0.54
45	1.09	1.06	1.04	1.32

**Table 3:** T_g_ values at different ageing times.

Specimen Type	T_g_ [°C]
0 days—no ageing—reference	116
1 day	114
3 days	111
7 days	106
45 days	98

**Table 4:** Compressive residual strength results (values reported in [MPa]).

	No Aged		1 Day		3 Days		7 Days		45 Days	
	Mean Value	Std. Dev.	Mean Value	Std. Dev.	Mean Value	Std. Dev.	Mean Value	Std. Dev.	Mean Value	Std. Dev.
**No Impact**	444.4	18.1	452.5	15.1	410.4	43.9	373.9	25.2	391.0	28.3
**NE**	425.6	24.5	400.6	31.3	405.2	23.5	419.8	13.7	383.7	20.4
**CI**	442.2	29.2	401.6	26.6	401.8	13.6	405.4	32.0	382.0	28.5

**Table 5:** Compressive residual strength variations (mean values, reported in [%]).

	No Aged	1 Day		3 Days		7 Days		45 Days	
	Impact Δσ	Impact Δσ	Ageing Δσ	Impact Δσ	Ageing Δσ.	Impact Δσ	Ageing Δσ	Impact Δσ	Ageing Δσ.
**No Impact**	-	-	1.8	-	−7.7	-	−15.9	-	−12.0
**NE**	−4.2	−11.5	−5.9	−1.3	−4.8	12.3	−1.4	−1.9	−9.9
**CI**	−0.5	−11.3	−9.2	−2.1	−9.1	8.4	−8.3	−2.3	−13.6

## Data Availability

The data presented in this study are available on request from the corresponding author.
